# Preparation, purification, and identification of novel antioxidant peptides derived from *Gracilariopsis lemaneiformis* protein hydrolysates

**DOI:** 10.3389/fnut.2022.971419

**Published:** 2022-07-22

**Authors:** Xiao Hu, Jing Liu, Jun Li, Yuqiong Song, Shengjun Chen, Shaobo Zhou, Xianqing Yang

**Affiliations:** ^1^Key Laboratory of Aquatic Product Processing, Ministry of Agriculture and Rural, South China Sea Fisheries Research Institute, Chinese Academy of Fishery Sciences, Guangzhou, China; ^2^Co-innovation Center of Jiangsu Marine Bio-industry Technology, Jiangsu Ocean University, Lianyungang, China; ^3^CAS Key Laboratory of Tropical Marine Bio-resources and Ecology, Guangdong Key Laboratory of Marine Materia Medica, RNAM Center for Marine Microbiology, South China Sea Institute of Oceanology, Chinese Academy of Sciences, Guangzhou, China; ^4^College of Food Science and Technology, Shanghai Ocean University, Shanghai, China; ^5^School of Life Sciences, Institute of Biomedical and Environmental Science and Technology, University of Bedfordshire, Luton, United Kingdom; ^6^Collaborative Innovation Center of Seafood Deep Processing, Dalian Polytechnic University, Dalian, China

**Keywords:** *Gracilaria lemaneiformis*, hydrolysis, antioxidant peptide, preparation, purification, structural identification

## Abstract

*Gracilariopsis lemaneiformis* (*G. lemaneiformis*) protein was hydrolyzed with alkaline protease to obtain antioxidant peptides. The enzymatic hydrolysis conditions were optimized through single-factor and orthogonal experiments. The results showed that the optimal process parameters were using 2% of alkaline protease, and substrate concentration of 1 g/100 mL and hydrolyzed 2 h at pH 8.0. Gel filtration chromatography and RP-HPLC were adopted for isolating and purifying the antioxidant peptides from the *G. lemaneiformis* protein hydrolysate (GLPH). Three novel antioxidant peptides were identified as LSPGEL (614.68 Da), VYFDR (698.76 Da), and PGPTY (533.57 Da) by nano-HPLC-MS/MS. The results of ABTS free radical scavenging rate demonstrated PGPTY exhibited the best antioxidant activity (IC_50_ = 0.24 mg/mL). Moreover, LSPGEL, VYFDR, and PGPTY were docked with Keap1, respectively. The molecular docking results suggested PGPTY had smaller docking energy and inhibition constants than the other two peptides. Finally, the cell viability assay evidenced the protective effect exerted by the antioxidant peptide on H_2_O_2_-induced oxidative damage. Above findings showed the potential of using antioxidant peptides from GLPH as antioxidants.

## Introduction

Bioactive peptides could be obtained from seaweed by direct extraction (physical extraction and chemical reagent extraction), enzymatic hydrolysis, microbial fermentation or chemical synthesis. They have special physiological regulation function to maintain human health (1). The global seaweed resources are extremely rich. Microalgae and phytoplankton have been paid more attention to because of their unique living habits and physiological structure. In addition, the research of macroalgae is gradually deepening (2). At present, studies have shown that the bioactive peptides in seaweed have anti-tumor proliferation (3), ACE inhibitory activity (4), scavenging free radicals (5), and other functional activities, but low toxicity (non-toxic) to normal cells. These activities indicate that algae enrich the source of bioactive peptides. Compared with the direct extraction, microbial fermentation, and chemical synthesis, enzymatic hydrolysis is one of the fastest, safest, and most controllable technologies for the preparation of bio-active peptides. It uses protease for catalyzing the peptide bond (ester bond or amide bond) hydrolysis to produce small molecule active substance. This specificity and hydrolysis conditions (pH, temperature, hydrolysis time, and enzyme dosage) affect peptide chain size, amino acid sequence and the amount of free amino acid, thus affecting the biological activity of the hydrolysate (6). The amino acid composition, sequence, length, total charge, and hydrophobicity of bioactive peptides will affect their activities (7).

The cells produce a lot of reactive oxygen species (ROS) during metabolism. Normally, the production and elimination of ROS will be in a state of balance. However, when ROS is produced too much, the balance will be broken, and the body will be in the state of oxidative stress and cause certain oxidative damage (8). Antioxidants exert significant impacts on reduction of oxidative damage. Synthetic antioxidants including butylated hydroxyanisole (BHA), butylated hydroxytoluene (BHT), tertiary butylhydroquinone (TBHQ), as well as propyl gallate are cheap and efficient. However, its potential toxicity and carcinogenicity limit its application in food industry. Therefore, more attention has been paid to natural antioxidants (9). Protein hydrolysates and peptides from seaweed have shown significant antioxidant activities and can be used as an alternative to synthetic antioxidants to reduce the potential risks of synthetic anti-oxidants (10, 11). For example, the peptide AF from the hydrolysates of *Pyropia columbina* displayed 1,1-diphenyl-2-picrylhydrazyl (DPPH) free radical (IC_50_ = 1.0 mg/mL) as well as 2, 2′-azino-bis (3-ethylbenzothiazoline-6-sulfonic acid) (ABTS) free radical (IC_50_ = 0.6 mg/mL) scavenging abilities (12). Besides, a peptide isolated from green algae *Halimeda macrobola* showed anticancer and antioxidant activities (13).

The red algae *Gracilaria lemaneiformis* (*G. lemaneiformis*) is widely distributed in marine environment, as China’s second largest seaweed cultivation (14). In recent years, many researchers have studied *G. lemaneiformis* polysaccharides, which have been proved to have antioxidant, antitumor, immunomodulatory, anti-aging and other activities (15–17), but there is little research on *G. lemaneiformis* protein. With the in-depth understanding of *G. lemaneiformis*, it is found that *G. lemaneiformis* not only has high protein content, but also has a high ratio of antioxidant amino acids such as Try, Phe, and Cys (accounting for about 22% of the total amino acid content). Therefore, it could be a good resource for preparation of antioxidant peptides.

In this research, alkaline protease was applied for hydrolyzing *G. lemaneiformis* protein for preparation of antioxidant peptides. Then the hydrolysis condition was optimized, and the degree of hydrolysis and ABTS free radical scavenging rate were used as evaluation indexes. The hydrolysates were further separated by gel-filtration chromatography and reversed-phase high-performance liquid chromatography. Using LC-MS/MS, the amino acid sequence of the purified peptide was identified. The identified antioxidant peptides were synthesized, followed by the determination of their antioxidant activities. The molecular docking and cell viability assay were further used to evaluate peptide antioxidant activity.

## Materials and methods

### Materials

*G. lemaneiformis* was purchased from Nan’ao Island (Shantou, Guangdong, China). Alkaline protease was provided by Hefei Bomei Biotechnology Co., Ltd. (Hefei, China). 1,1-diphenyl-2-picrylhy-drazyl (DPPH), 2, 2′-azino-bis (3-ethylbenzothiazoline-6-sulfonic acid) (ABTS) and tripyridyltriazine (TPTZ) were provided by Sigma-Aldrich Co., Ltd. (St. Louis, MO, United States). HepG2 was provided by Chinese Academy of Sciences. DMEM medium and fetal bovine serum (FBS) were supported by Gibco Co., Ltd. (Guangzhou, China). Superoxide dismutase (SOD) activity detection kit and malondialdehyde (MDA) assay kit were provided by Beijing Solarbio Science and Technology Co., Ltd. (Beijing, China).

### Preparation of *Gracilaria lemaneiformis* protein

In this study, the dried *G. lemaneiformis* powder was immersed in 0.2 M NaOH at the proportion of 1:24 (w/v). Next, in this study, the mixtures were employed in *G. lemaneiformis* protein extraction using ultrasonic assisted alkali extraction and acid precipitation. Ultrasonic treatment was implemented at 30°C in 70 min at a power of 482 W, and the frequency parameter is set to 20 kHz. We centrifuged later solution at 11,000 *g* in 15 min at 4°C. In addition, the pH in supernatant was set to 4.5, and centrifugated at 11,000 *g* for 15 min at 4°C. Then, the collected precipitate was dialyzed and lyophilized. Finally, the obtained *G. lemaneiformis* protein was preserved at −20°C for future application.

### Preparation of *Gracilariopsis lemaneiformis* protein hydrolysate

*G. lemaneiformis* protein were dissolved into ultrapure water for preparation of solutions with different protein concentration. The protein solution was hydrolyzed with alkaline protease at their optimal condition. Followed by centrifugation of protein hydrolysates at 11,000 *g* in 15 min at 4°C, the reaction ceased after heating at 100°C for 20 min. Next, in order to perform the analysis, the obtained supernatant received freeze drying treatment and was preserved at −20°C. The degree of hydrolysis was calculated from the ratio of α-amino nitrogen to total nitrogen (the amino nitrogen content was measure by the formaldehyde titration method, and the total nitrogen content as well as protein concentration were measured by the Kjeldahl method).

#### Single-factor experimental design

The digestion conditions in single-factor experiments included enzyme dosage (1, 2, 3, 4, 5, and 6%, mass ratio of enzyme to substrate), hydrolysis time (1, 2, 4, 6, 8, and 10 h), substrate concentration (0.5, 1, 1.5, 2, 2.5, and 3 g/100 mL), pH (7, 7.5, 8, 8.5, and 9), and their effects (digestion temperature was kept at 50°C) on the degree of hydrolysis and ABTS radical scavenging activity were investigated, respectively.

#### Orthogonal experiment design

In view of the findings obtained from single-factor experiment, the range and central point value of substrate concentration (A), the enzyme dosage (B), hydrolysis time (C) and pH (D) were determined. Then the enzyme digestion temperature was kept constant set at 50°C. The processing parameters were optimized using orthogonal experiment and every chosen variable was coded at three levels (1, 2, 3) (Table 1). Besides, the ABTS radical scavenging activity in the enzymatic hydrolysate formed in optimal conditions was measured in order to verify the predictive enzymatic hydrolysis conditions model.

**TABLE 1:** Table of orthogonal experiment factors.

Factor	Lever		
	1	2	3
A (substrate concentration)/%	0.5	1	1.5
B (enzyme dosage)/%	2	3	4
C (enzymolysis time)/h	1	2	3
D (pH)	7.5	8	8.5

### Antioxidant activity

#### Diphenyl-2-picrylhy-drazyl radical scavenging activity

The DPPH radical scavenging activity was determined according to the method of Hatamnia et al. (18) after slight modifications. 0.1 ml of samples consisting of ultrapure water were placed in centrifugal tube, and 100 μL of an ethanolic solution of 0.1 mM DPPH was added. Next, the mixture was mixed completely, followed by incubation at room temperature for half an hour in darkness. Mixture absorbance was tested as 517 nm. The DPPH solution was substituted with ethanolic in the blank.

DPPH radical scavenging activity could be expressed as:


(1) $$\text{DPPH}\cdot \text{scavenging}\text{activity}\left(\%\right)=\left(1-\frac{\text{A}-{\text{A}}_{b}}{{\text{A}}_{0}}\right)\times 100\%$$

where A means the absorbance for the sample, A_*b*_ means the absorbance for the control (containing DPPH solution, excluding the sample), and A_0_ means the absorbance for the blank.

#### Azino-bis (3-ethylbenzothiazoline-6-sulfonic acid) radical scavenging activity

In this study, ABTS radical scavenging activity was tested with the technique by Chi et al. (19) after slight modifications. ABTS solution was fabricated through mixing 14.8 mM ABTS stock solution and 5.2 mM potassium persulphate. The mixture received 12-h reaction in darkness. The ABTS^+^ working solution was diluted in ethanolic without water to acquire the absorbance of 1.1 ± 0.02 at 734 nm. A total of 50 μL sample at varying concentrations was blended with 950 μL diluted ABTS^+^ working solution. Besides, the mixture was mixed completely, followed by incubation at chamber temperature for 2 h in darkness. Mixture absorbance was calculated at 734 nm. ABTS radical scavenging activity was measured by:


(2) $$\text{ABTS}\text{radical}\text{scavenging}\text{activity}\left(\%\right)=\left(1-\frac{{\text{A}}_{\mathrm{sample}}}{{\text{A}}_{\mathrm{control}}}\right)\times 100\%$$

#### The ferric reducing antioxidant power

Besides, the ferric reducing antioxidant power (FRAP) was calculated following the procedure by Sato et al. (20) after slight modifications. FRAP working solution [0.3 M acetate buffer (pH 3.6), 0.01 M, 2,4,6-tripyridine-s-triazine (TPTZ), 0.02 M FeCl_3_ 6H_2_O, 10:1:1, were prepared and used now]. According to the absorbance after reaction of FeSO_4_ standard solution at varying concentrations (0.1, 0.2, 0.4, 0.6, 0.8, and 1.0 mM) and FRAP working solution, the standard curve (y = 0.2323x + 0.0012, *R*^2^ = 0.996, where y indicates the absorbance value; x is the concentration of FeSO_4_, mM) was founded. 1.8 mL of FRAP working solution was added into 0.2 mL 2 mg/mL of the different sample concentrations in the centrifugal tube. Next, the mixture was mingled completely and cultured at 37°C in half an hour. Mixture absorbance was calculated at 593 nm. Meanwhile, 0.1 mL distilled water was used as blank. FRAP was measured with the following equation:


(3) FRAPvalue(μg/mL)=C×N×M

C represents the Fe^2+^ equivalent (mM) obtained by substituting the absorbance of the sample after deducting the blank into the regression equation, N represents the dilution multiple of the sample, M represents the molar mass of FeSO_4_ (g/mol).

### Purification of antioxidant peptide from *Gracilariopsis lemaneiformis* protein hydrolysate

Firstly, the GLPH solution (filtered by 0.22 μm microporous membrane) was fractionated on a Sephadex G-25 gel filtration column (1.6 × 50 cm) with the flow rate of 0.5 mL/min. In the meanwhile, the column was eluted in ultrapure water. Every eluate was gathered for monitoring at 214 nm, and four fractions (A–D) were gathered and lyophilized. The highest antioxidant activity fraction C was further separated using RP-HPLC. The peptide fraction was filtered through a microporous membrane (0.22 μm) before being loaded into Athena C18 (5 μm, 4.6 × 250 mm). Besides, the mobile phases of gradient elution included eluent A including 0.1% concentration of TFA in ultrapure water (v/v) and eluent B including 0.1% concentration of TFA in acetonitrile. In addition, the gradient elution program was 0–5 min, 0% A; 5–25 min, 0–50% A; 25–30 min, 50–100% A. The eluate was monitored at 220 nm, and three peptides (C1–C3) were isolated and then lyophilized.

### Identification of the amino acid sequence

The fraction C1 having the highest antioxidant activity was further analyzed the amino acid sequence by nano-HPLC-MS/MS on a Q Exactive Plus mass spectrometry (Thermo Fisher Scientific, Waltham, MA, United States). We loaded 9 μL sample into a chromatographic analytical column (Acclaim PepMap C18, 75 μm × 15 cm) at a flow rate of 300 nL/min. Elution conditions included mobile phase A (0.1% formic acid in water); mobile phase B (0.1% formic acid in ACN); column temperature (40°C); linear elution program (0–30 min, 8–30% B). Besides, mass spectra were documented within an m/z scope of 100–1,500. In addition, we had the electrospray voltage of 2 kV. Tandem mass spectra were processed by PEAKS Studio version X+ (Bioinformatics Solutions Inc., Waterloo, ON, Canada).

### Peptide synthesis

Synthesis of identified antioxidant peptides followed the solid-phase technique of GL Biochemistry Co., Ltd. (Shanghai, China). Besides, the resulting peptides had a purity over 95% (w/w) measured using high-performance liquid chromatography at mobile phase A, 0.1% TFA in 100% acetonitrile; mobile phase B, 0.1% TFA in 100% water; flow rate, 1 mL/min; column, Kromasil-C18, 5 μm particle size, 4.6 × 250 mm. Using the MS spectrum, molecular masses of synthesized peptides were measured. The ABTS radical scavenging activity of synthesized peptides with varying concentrations (0–1 mg/mL) was measured as mentioned previously. Using non-linear regression analysis, the half inhibitory concentration (IC_50_) of the peptide was measured.

### Molecular docking analysis

In this study, the molecular docking in the identified peptides with Keap1 was performed using the procedure by Han et al. (21). The semi flexible docking of Keap1 with antioxidant peptide was calculated by molecular docking software AutoDock 4.2. Before docking, we downloaded the crystal structure of Keap1 (PDB ID: 2FUL) from the PDB database. Simultaneously, water molecules and Nrf2 16-mer were removed by PyMOL software to obtain the receptor molecules to be docking. Adding polar hydrogen to the receptor molecule, calculate the charge and add the protein type. The 3D structure of small molecule ligands was obtained in Chem3D software and the energy was minimized. In autodock, small molecule ligands can be detected. The central coordinates of the docking range were set as the active site of Keap1 (x: 5; y: 9; z: 1) (22). At the same time, and a reaction constraint box with a spacing of 0.375 Å was established. Lamarck genetic algorithm is used to optimize the docking energy. In the cluster analysis of conformational energy, the tolerance of root mean square deviation is 2.0 Å. The interaction between antioxidant peptides from *G. lemaneiformis* and Keap1 protein was analyzed by discovery studio 2020 client.

### H_2_O_2_-induced oxidative stress HepG2 cells model

H_2_O_2_-induced oxidative damage model was established, and the influence of the antioxidant peptide on oxidative damage was evaluated by measuring cell viability, SOD activity and MDA content. HepG2 cells suspension was absorbed and seeded on 96-well plates with the density of 1 × 104 cells/well, followed by cultivation under a humidified atmosphere (37°C, 5% CO_2_) *via* DEME medium (with 10% FBS and 1% penicil-lin-streptomycin). After 24-h cultivation of HepG2, the cells were categorized into control and treatment groups. In addition, the control group was added with 100 μL culture medium and cultured for 4h. Besides, the treatment group (peptide-treated group) was pre-pared through supplementing 100 μL of 100 μg/mL of GSH or PGPTY (DMEM dissolved) and cultured for 4 h. Next, 10 μL H_2_O_2_ (800 μM) was introduced to every well, followed by incubation for 2 h. Besides, the treatment group (H_2_O_2_-treated group) was added with 100 μL culture medium and cultured for 4 h. Then, 10 μL H_2_O_2_ (800 μM) was introduced, which was also cultured for 2 h. Light density value at 595 nm was calculated, and cell viability (%) was computed as below:


(4) $$\text{cell}\text{viability}\;(\%)=\frac{{\text{A}}_{\mathrm{treatment}\mathrm{group}}}{{\text{A}}_{\mathrm{control}\mathrm{group}}}\times 100\%$$

Where A_*treatment group*_ indicates the absorbance in treatment group, A_*control group*_ denotes the absorbance in control group.

SOD activity and MDA content were determined using SOD kit and MDA kit (Beijing Solarbio Science and Technology Co., Ltd.).

### Statistical analysis

Through one-way analysis of variance (ANOVA) on the IBM SPSS statistics 25.0 software, Duncan’s *t*-tests were conducted. It was shown that differences showed statistical significance at *p* < 0.05.

## Results and discussion

### Determination of single-factor experiment

**FIGURE 1:**
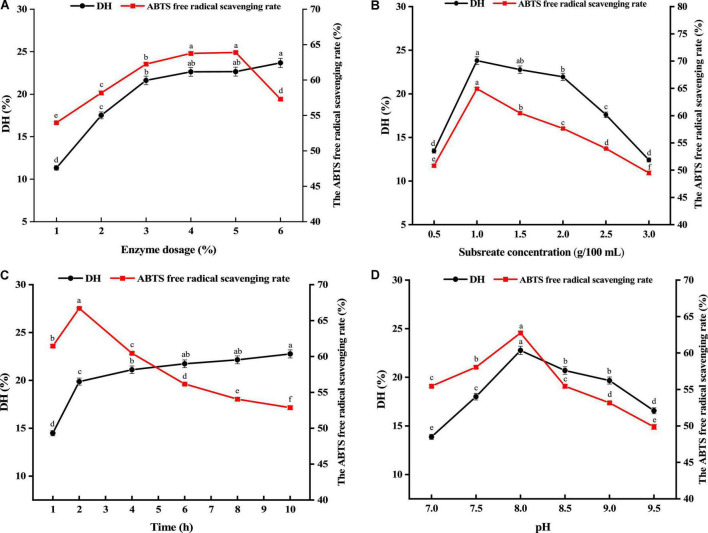
Effects of enzyme dosage **(A)**, substrate concentration **(B)**, hydrolysis time **(C)**, and pH **(D)** on the degree of hydrolysis (DH) and ABTS free radical scavenging rate. Different lower cases above the error bar suggest obvious differences of groups (*p* < 0.05).

The *G. lemaneiformis* protein was hydrolyzed by alkaline protease. The effects of enzyme dosage, enzyme hydrolysis time, substrate concentration, pH on the degree of hydrolysis and the ABTS free radical scavenging rate of protein hydrolysates (2 mg/mL) were investigated by single factor experiment. Optimization of enzymatic hydrolysis conditions (such as substrate concentration, enzyme dosage, enzymatic hydrolysis time, and pH) is crucial for the production of hydrolyzed proteins with desired functional properties (23). Enzymatic hydrolysis program was carried on with different enzyme dosage from 1 to 6%, and other hydrolysis conditions were fixed with temperature of 50°C, enzyme hydrolysis time of 4h, substrate concentration of 1 g/100 mL and pH 8.0. As shown in Figure 1A, the degree of hydrolysis and the ABTS free radical scavenging rate rapidly enhanced from 1 to 3%. After enzyme dosage more than 3%, the degree of hydrolysis and the ABTS free radical scavenging rate increased slightly. Nevertheless, the ABTS free radical scavenging rate appeared a downward trend with enzyme dosage more than 5%. Moreover, no significant difference was found in hydrolysis degree and ABTS free radical scavenging rate between 4 and 5%. The principle of reagent saving and antioxidant activity were used as the main criteria, and the 2–4% of enzyme dosage was chosen as the subsequent optimized experimental conditions.

As an important factor affecting the activity and degree of hydrolysis of enzymatic hydrolysis products, the optimal conditions of substrate concentration were also explored varying from 0.5 to 3 g/100 mL. Figure 1B exhibits the impact of substrate concentration on hydrolysis degree and ABTS free radical scavenging rate. As substrate concentration grew to 1 g/100 mL, hydrolysis degree and ABTS radical scavenging rate both shown an upward trend, and the maximum antioxidant activity was reached at 1 g/100 mL. As substrate concentration continually increases, hydrolysis degree and ABTS radical scavenging rate gradually decrease. The reason for this may be that too high substrate concentration will cause the solution to be too viscous, slow molecular diffusion, and cause substrate inhibition and enzyme inactivation. Thus, substrate concentrations ranged from 0.5 to 1.5 g/100 mL were chosen as the subsequent optimized experimental conditions.

The impact of enzyme hydrolysis time on hydrolysis degree and ABTS free radical scavenging rate was within a range of 1–10h. According to Figure 1C, as nzymatic hydrolysis time increased from 1 to 2 h, hydrolysis degree and ABTS free radical scavenging rate showed a fast increase. As enzymatic hydrolysis time enhanced, hydrolysis degree presented a slow increase, but ABTS continually decreased. It was possibly because the prolonged hydrolysis time made hydrolysis more complete. But excessive hydrolysis could produce more amino acids, resulting the decreased antioxidant activity. In addition, the highest value of the ABTS free radical scavenging rate (66.71%) was found at enzymatic hydrolysis time of 2 h. In order to make the peptides obtained in the subsequent experiments have better biological activity, the enzymatic hydrolysis time from 1 to 3 h was selected as the subsequent optimized experimental conditions.

The effect of pH on both hydrolysis degree and ABTS free radical scavenging rate was within a range of 7.0–9.5. Figure 1D shows the results. As pH increased, hydrolysis degree and ABTS free radical scavenging rate showed a fast increase. As pH approached 8, hydrolysis degree and ABTS free radical scavenging rate reached the maximum. Hydrolysis degree and ABTS radical scavenging rate progressively reduced as pH continually increased. Therefore, pH ranged from 7.5 to 8.5 was selected.

### Orthogonal experimental analysis

By performing the single-factor experiment, the orthogonal experiment was performed using the 2.3.2 method, in which ABTS free radical scavenging rate served as an evaluation index. The experiment results and analysis are shown in Table 2. The extreme value R shows that the primary and secondary order of the influence of different factors on the scavenging rate of ABTS free radicals within the experimental range is A (substrate concentration) > B (enzyme dosage) > C (hydrolysis time) > D (pH). The theoretical optimal combination of each factor is A2B1C2D2. The analysis of variance of orthogonal experiment was shown in Table 3. The impacts of A, B, C, and D on the clearance rate of ABTS radical scavenging rate were significant (*p* < 0.05). Finally, the optimal process parameters were determined (enzyme dosage of 2%, hydrolysis time of 2 h, substrate concentration of 1 g/100 mL and pH at 8.0).

**TABLE 2:** Orthogonal experimental analysis.

Experiment number	A	B	C	D	ABTS free radical scavenging rate (%)
1	1	1	1	1	61.16 ± 0.54
2	1	2	2	2	62.96 ± 0.42
3	1	3	3	3	58.97 ± 0.35
4	2	1	2	3	72.57 ± 0.49
5	2	2	3	1	64.81 ± 0.19
6	2	3	1	2	69.03 ± 0.10
7	3	1	3	2	70.83 ± 0.58
8	3	2	1	3	61.78 ± 0.35
9	3	3	2	1	66.78 ± 0.29
K1	183.08	204.55	191.96	192.75	
K2	206.41	189.54	202.30	202.81	
K3	199.38	194.77	194.60	193.31	
k1	61.03	68.18	63.99	64.25	
k2	68.80	63.18	67.43	67.60	
k3	66.46	64.92	64.87	64.44	
Extreme value R	23.33	15.01	10.34	10.06	
Order of the factor	A > B > C > D				
Optimum combination	A_2_	B_1_	C_2_	D_2_	

**TABLE 3:** Analysis of orthogonal experimental variance.

Sources	Type III sum of squares	*df*	Mean square	*F*	Significance
Modified model	524.20	8	65.53	412.00	<0.001
Intercept	115589.35	1	115589.35	726798.52	<0.001
A	286.43	2	143.21	900.50	<0.001
B	116.08	2	58.04	364.95	< 0.001
C	57.75	2	28.88	181.57	<0.001
D	63.93	2	31.97	201.00	<0.001
Error	2.86	18	0.16		
Sum	116116.41	27			
Revised total	527.06	26			

### Purification of antioxidant peptides From *Gracilaria lemaneiformis* protein hydrolysate

**FIGURE 2:**
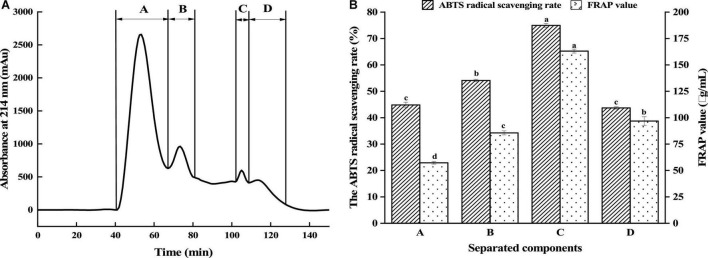
Separation of GLPH by gel filtration chromatography **(A)** and antioxidant activity of the separated fractions **(B)**. Different lowercases above the error bar suggest obvious differences in antioxidant activity of separated components (*p* < 0.05).

Peptide size is among the influencing factors of its antioxidant activity. Gel filtration chromatography and RP-HPLC are commonly used to isolate and enrich peptides (24, 25). The antioxidant peptides from GLPH were isolated using the Sephadex G-25 gel filtration column according to the differences of molecular dimensions. Figure 2A displayed that GLPH was separated into four fractions (A, B, C, and D). Every fraction was gathered for freeze drying, to investigate its antioxidant activity. As shown in Figure 2B, the fraction C exhibited the strongest antioxidant activity. Its ABTS free radical scavenging rate and FRAP value of the fraction C (2 mg/mL) was 74.95% and 163.08 μg/mL, respectively. Therefore, the fraction C was chosen in further separation and purification.

**FIGURE 3:**
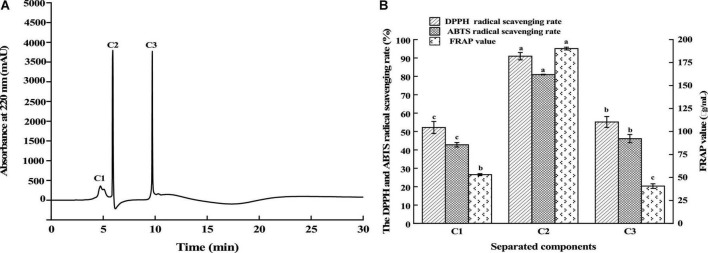
Separation of the fraction C by RP-HPLC **(A)** and the antioxidant activity of fractions **(B)**. Different lowercases above the error bar suggest obvious differences in antioxidant activity of separated components (*p* < 0.05).

Fraction C was isolated using RP-HPLC on an Agilent ZORBAX XDB-C18 (21.2 × 150 mm, 5 μm) column. According to Figure 3A, the elution consisted of three major peaks (C1, C2, and C3). Their antioxidant activities are displayed in Figure 3B. It was found that the fraction C2 (2 mg/mL) had the highest DPPH and ABTS free radical scavenging rate and FRAP value, which were 90.96, 80.95, and 190.17 μg/mL, respectively. Using nano-HPLC-MS/MS, the amino acid sequences of antioxidant peptides in fraction C2 were further measured.

### Identification of antioxidant peptides

**FIGURE 4:**
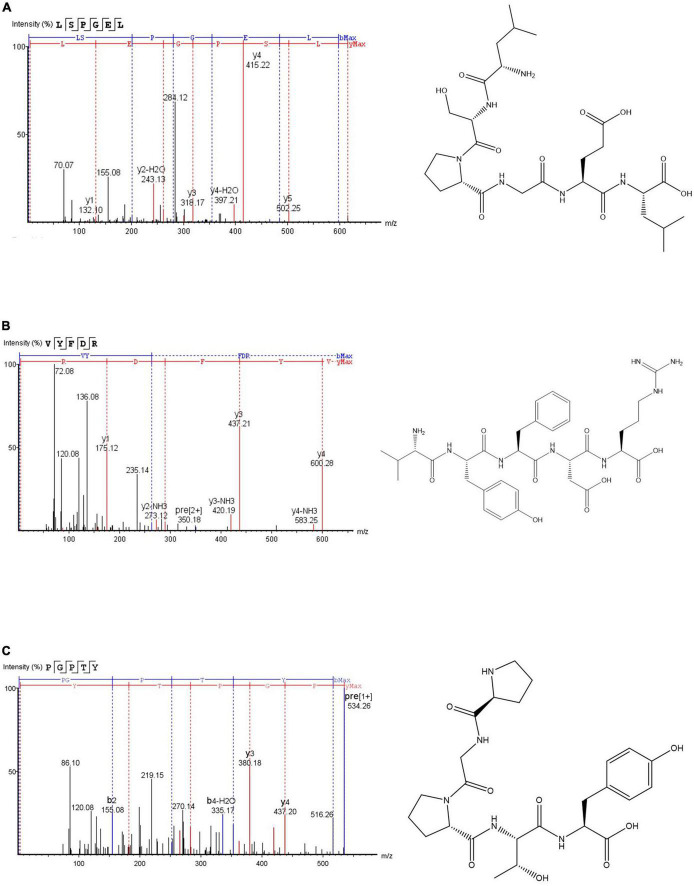
Secondary mass spectra and chemical structures of LSPGEL **(A)**, VYFDR **(B)**, and PGPTY **(C)**.

According to Figure 4, the three antioxidant peptides were identified as Leu-Ser-Pro-Gly-Glu-Leu (LSPGEL), Val-Tyr-Phe-Asp-Arg (VYFDR) and Pro-Gly-Pro-Thr-Tyr (PGPTY) with molecular weight of 614.68, 698.76, and 533.57 Da, respectively. The molecular weight of most antioxidant peptides is lower than 1,000 Da. This could be attributed to the peptides with lower molecular weight are more likely to have interactions with target free radicals to end the chain reaction, and they usually have stronger antioxidant activity than that of the peptides with higher molecular weight (26). As shown in Table 4, the corresponding proteins of the antioxidant peptides were verified, and the peptides of LSPGEL, VYFDR and PGPTY were found derived from allophycocyanin α chain (18th–23th), phycocyanin beta subunit (162th–166th), and phycocyanin α subunit (70th–74th), respectively. Synthesis of the identified peptides was performed to verify antioxidant activity. PGPTY had clearly higher antioxidant activity compared with LSPGEL and VYFDR.

**TABLE 4:** Sequencing results of the antioxidant peptides obtained from *G. lemaneiformis*.

Protein source	Sequence	MW (Da)	ABTS free radical scavenging activity (IC_50_, mg/mL)
Allophycocyanin α chain (18th–23th)	LSPGEL	614.68	5.35
Phycocyanin β subunit (162th–166th)	VYFDR	698.76	1.21
Phycocyanin α subunit (70th–74th)	PGPTY	533.57	0.24

### Molecular docking of identified peptides with Keap1

**FIGURE 5:**
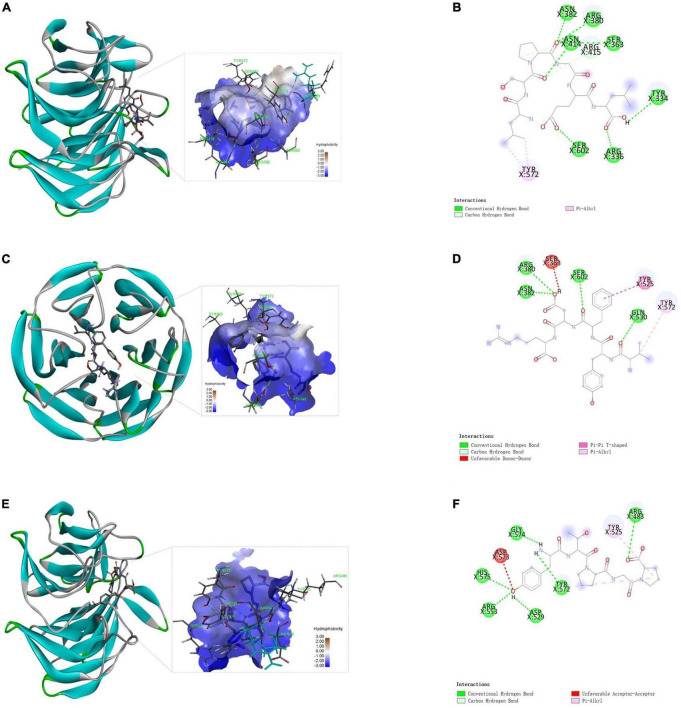
Docking results of Keap1 and the peptides. Three-dimensional **(A)** and two-dimensional **(B)** structure diagrams of LSPGEL and Keap1; three-dimensional **(C)** and two-dimensional **(D)** structure diagrams of VYFDR and Keap1; three-dimensional **(E)** and two-dimensional **(F)** structure diagrams of PGPTY and Keap1.

Keap1-Nrf2 pathway is the most important regulator of oxidative stress induced by various exogenous and endogenous factors (27). Keap1 (Kelch-like ECH-associated protein 1) could bind to Nrf2 (nuclear factor erythroid 2-related factor 2) *via* its Kelch domains, leading to a degradation of Nrf2 (28). However, the accumulation of Nrf2 is very important for the expression of a series of phase II detoxication enzymes such as superoxide dismutase (SOD) in the cells (29). Therefore, the external molecules (like peptides) able to bind to Keap1 could prohibit Keap1-Nrf2 interaction and increase cell resistance to oxidative stress. The docking of antioxidant peptide with Keap1 by Autodock 4.2 will help to further analyze the anti-oxidation mechanism. As shown in Figures 5A,B, LSPGEL forms seven hydrogen bonds with ASN382, ARG380, ASN414, SER363, TYR334, SER602, and ARG336 of Keap1, forms hydrocarbon bond with ARG415, and forms PI alkyl with TYR572, with docking energy of −3.88 kcal/mol and inhibition constant of 1.43 mM. As shown in Figures 5C,D, four hydrogen bonds were formed between VYFDR and Keap1’s ARG380, ASN382, SER602, and GLN530, with docking energy of −3.49 kcal/mol and inhibition constant of 2.79 mM. According to Figures 5E,F, six hydrogen bonds were formed between PGPTY and keyap1’s GLY574, HIS575, ARG553, ASP529, TYR572, ARG483, with docking energy of −4.49 kcal/mol and inhibition constant of 0.51 mM. The results suggested that the PGPTY docked with Keap1 was easier than the other two peptides, which might be the cause of the PGPTY had the higher antioxidant activity.

Active peptides are mainly combined with macromolecules through hydrogen bond interaction, van der Waals force and electrostatic interaction. Besides, hydrogen bond interaction is particularly important, and can play a role in stabilizing docking complex. Keap1 is a key component of Nrf2 regulation that plays a part in cytoplasm. The peptide LSPGEL and VYFDR occupied the amino acid residues ARG380 and ASN382, while the peptide PGPTY occupied the amino acid residues ARG483. These amino acid residues are very important for the Keap1 in the Keap1-Nrf2 pathway. Thus, the antioxidant peptide from *G. lemaneiformis* has a favorable effect on the cell oxidative stress and exogenous damage.

### Cytoprotective effects against cell damage induced by H_2_O_2_

**FIGURE 6:**
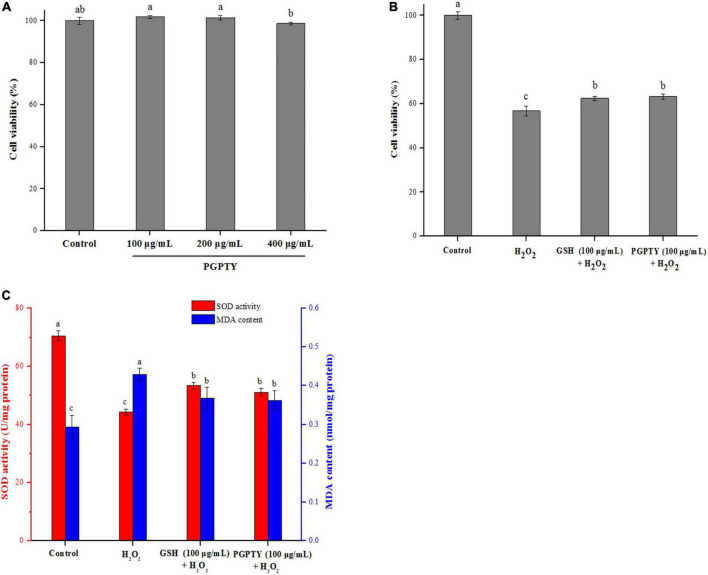
Protective impacts of antioxidant peptide against H_2_O_2_-induced stress damage in HepG2 cells. **(A)** Cytotoxicity of PGPTY at varying concentrations in HepG2 cells. **(B)** Cell viability of HepG2 cells treated with H_2_O_2_, GSH + H_2_O_2_ and PGPTY + H_2_O_2_. **(C)** SOD activity and MDA con-tent of HepG2 cells treated with H_2_O_2_, GSH + H_2_O_2_ and PGPTY + H_2_O_2_. Different lowercases above the error bar suggest obvious differences of groups (*p* < 0.05).

By adopting the H_2_O_2_-induced oxidative stress cell model, the antioxidant activity of PGPTY was assessed. Figure 6A demonstrates that different concentrations (100, 200, 400 μg/mL) of PGPTY had no significant (*p > 0.05*) toxicity impact on HepG2 cells in contrast to the control group. With the purpose of exploring the protective impact of PGPTY on H_2_O_2_-induced cell damage, PGPTY (100 μg/mL) and positive control glutathione (100 μg/mL) were pretreated and then exposed to 800 μM of H_2_O_2_, respectively. As illustrated in Figure 6B, the cell viability of HepG2 cells cultured with H_2_O_2_ (800 μM) was significantly decreased (*p* < 0.05) to 56.71%, indicating that the oxidative damage model for HepG2 cells was successfully constructed. When HepG2 cells were pretreated with PGPTY or GSH and then treated with H_2_O_2_, the cell viability was significantly enhanced (*p < 0.05*). Research showed PGPTY and GSH could both alleviate the damage of HepG2 cells induced by H_2_O_2_, and the cytoprotective effect of the PGPTY was as well as that of the GSH.

For illustrating the protective mechanism of PGPTY against H_2_O_2_-induced oxidative stress injury in HepG2 cells, SOD activity and MDA content in HepG2 cells were studied. Besides, the antioxidant enzymes (such as SOD) in cell are very important for the cell against oxidative stress damage, and the cell with high antioxidant enzyme activity could have good antioxidation ability (30, 31). Malondialdehyde (MDA) could be produced from the degradation of cell membrane induced by lipid peroxidation, and the reduced content of MDA in the cell could reflect the alleviated oxidative damage (32, 33). As shown in Figure 6C, when HepG2 cells were processed using 800 μM H_2_O_2_ in 2h, SOD activity in HepG2 cells was drastically decreased and MDA content obviously ascended. However, the PGPTY protective group had obviously higher SOD activity (*p* < 0.05) in relative to the H_2_O_2_-injured group, indicating that the capacity of PGPTY to prevent H_2_O_2_-induced oxidative damage on HepG2 cells was in part associated with its ability to enhance antioxidant enzyme (such as SOD) activity in HepG2 cells. Besides, the MDA content in PGPTY-pretreated HepG2 cells was notably lower (*p* < 0.05) when compared with that of H_2_O_2_-injured group, implying cell membrane could be protected by the peptide from H_2_O_2_-induced degradation or damage. Similar regulation effect of SOD and MDA in the H_2_O_2_-injured HepG2 cells was also observed in the GSH-pretreated group, and no significant (*p* > 0.05) difference was reported between groups.

## Conclusion

In the present study, *G. lemaneiformi*s protein was hydrolyzed using alkaline protease to obtain antioxidant peptides. The enzymatic hydrolysis conditions were optimized through single-factor and orthogonal experiments, and the optimal technological parameters were obtained (2% enzyme, 2 h hydrolysis, 1 g/100 mL of substrate concentration, and pH at 8.0). The antioxidant peptides in the GLPH were then separated and purified by using gel filtration chromatography and RP-HPLC, and then three novel antioxidant peptides were identified as LSPGEL, VYFDR, and PGPTY. Compared with the other two peptides, PGPTY possessed higher antioxidant activity and could dock with Keap1 more easily. The cell viability assay indicated that protective effect of PGPTY on H_2_O_2_-induced HepG2 cells damage was as good as that of the GSH. Therefore, it is possible to produce peptides with potent antioxidant activity from *G. lemaneiformis* protein by proteolytic hydrolysis and purification.

## Data availability statement

The original contributions presented in this study are included in the article/supplementary material, further inquiries can be directed to the corresponding author/s.

## Author contributions

XH and JLi: conceptualization. XH, YS, and SC: data curation. XH, JLiu, and SC: formal analysis. XY: funding acquisition. XH and JLiu: investigation and writing—original draft. XH, SZ, and SC: methodology. XY: resources. JLi: supervision. XH, JLiu, and SZ: validation. JLi: writing—review and editing. All authors have read and agreed to the published version of the manuscript.

## Conflict of interest

The authors declare that the research was conducted in the absence of any commercial or financial relationships that could be construed as a potential conflict of interest.

## Publisher’s note

All claims expressed in this article are solely those of the authors and do not necessarily represent those of their affiliated organizations, or those of the publisher, the editors and the reviewers. Any product that may be evaluated in this article, or claim that may be made by its manufacturer, is not guaranteed or endorsed by the publisher.
